# FLT3-ITD Compared with DNMT3A R882 Mutation Is a More Powerful Independent Inferior Prognostic Factor in Adult Acute Myeloid Leukemia Patients After Allogeneic Hematopoietic Stem Cell Transplantation: A Retrospective Cohort Study

**DOI:** 10.4274/tjh.2018.0017

**Published:** 2018-08-05

**Authors:** Majid Teremmahi Ardestani, Ahmad Kazemi, Bahram Chahardouli, Saeed Mohammadi, Mohsen Nikbakht, Shahrbano Rostami, Mahdi Jalili, Mohammad Vaezi, Kamran Alimoghaddam, Ardeshir Ghavamzadeh

**Affiliations:** 1 Iran University of Medical Sciences, School of Allied Medical Sciences, Department of Hematology, Tehran, Iran; 2 Tehran University of Medical Sciences, Cell Therapy and Hematopoietic Stem Cell Transplantation Research Center; Hematology, Oncology and Stem Cell Transplantation Research Center, Tehran, Iran

**Keywords:** Allogeneic hematopoietic stem cell transplantation, Acute myeloid leukemia, DNMT3A R882, FLT3-ITD

## Abstract

**Objective:**

This study aimed to evaluate DNMT3A exon 23 mutations and their prognostic impacts in the presence of NPM1 and FLT3 mutations in acute myeloid leukemia (AML) patients who underwent allogeneic hematopoietic stem cell transplantation (HSCT).

**Materials and Methods:**

This study comprised 128 adult AML patients referred to the Hematology-Oncology and Stem Cell Research Center of Shariati Hospital. NPM1 and FLT3-ITD mutations were detected by fragment analysis. For DNMT3A exon 23 mutation analysis, we used Sanger sequencing. Overall survival (OS) and relapse-free survival (RFS) curves were estimated by the Kaplan-Meier method and the log-rank test was used to calculate differences between groups.

**Results:**

The prevalence of DNMT3A exon 23 mutations was 15.6% and hotspot region R882 mutations were prominent. RFS and OS were compared in patients with and without DNMT3A exon 23 mutations using univariate analysis and there was no significant difference between these groups of patients. On the contrary, the FLT3-ITD mutation significantly reduced the OS (p=0.009) and RFS (p=0.006) in AML patients after allogeneic HSCT. In the next step, patients with AML were divided into four groups regarding FLT3-ITD and DNMT3A mutations. Patients with DNMT3A R882mut/FLT3-ITDpos had the worst OS and RFS. These results indicate that DNMT3A mutations alone do not affect the clinical outcomes of AML patients undergoing allogeneic HSCT, but when accompanied by FLT3-ITD mutations, the OS was significantly reduced (5-year OS 0% for DNMT3A R882mut/FLT3-ITDpos patients vs. 62% DNMT3A R882wt/FLT3-ITDneg, p=0.025) and the relapse rate increased.

**Conclusion:**

It can be deduced that DNMT3A R882mut/FLT3-ITDpos is an unfavorable prognostic factor in AML patients even after allogeneic HSCT.

## Abstract (tr)

**Amaç:**

Bu çalışmada, allogeneik hematopoetik kök hücre nakli (HKHN) geçiren akut myeloid lösemi (AML) hastalarında NPM1 ve FLT3 mutasyonlarının varlığında, DNMT3A ekzon 23 mutasyonlarının prognostik etkilerinin değerlendirmesi amaçlanmaktadır.

**Gereç ve Yöntemler:**

Bu çalışma Shariati Hastanesi Hematoloji-Onkoloji ve Kök Hücre Araştırma Merkezi’ne başvuran 128 erişkin AML hastasını kapsamaktadır. NPM1 ve FLT3-ITD mutasyonları, fragman analizi ile tespit edilmiştir. DNMT3A ekzon 23 mutasyon analizi için Sanger dizi analizi kullanılmıştır. Genel sağkalım (OS) ve relapsız sağkalım (RFS) eğrileri için Kaplan-Meier yöntemi ve gruplar arası farklılıkları hesaplamak için log-rank testi kullanılmıştır.

**Bulgular:**

DNMT3A ekzon 23 mutasyonlarının prevalansı %15,6 olarak bulunmuştur ve bunların içinde sıcak bölge R882 mutasyonları öne çıkmaktadır. Tek değişkenli analiz kullanılarak DNMT3A ekzon 23 mutasyonları olan ve olmayan hastalarda RFS ve OS karşılaştırılmış ve bu hasta grupları arasında anlamlı fark bulunmamıştır. Aksine, FLT3-ITD mutasyonu taşıyan ve allogeneik HKHN geçiren AML hastalarında OS (p=0,009) ve RFS (p=0,006) değerleri anlamlı derecede düşmüş olarak gözlenmiştir. Bir sonraki adımda, AML hastaları FLT3-ITD ve DNMT3A mutasyonları açısından dört gruba ayrılmıştır ve DNMT3A R882mut/FLT3-ITD mutasyonu taşıyan hastalar en kötü OS ve RFS’ye sahip olarak bulunmuştur. Bu sonuçlar, DNMT3A mutasyonlarının tek başına allogenik HKHN yapılan AML hastalarının klinik sonuçlarını etkilemediğini işaret etmektedir, ancak FLT3-ITD mutasyonları eşlik ettiği zaman, OS’nin önemli ölçüde azaldığı (DNMT3A R882mut/FLT3-FLT3-ITD pozitif hastalarda 5 yıllık OS %0 ve DNMT3A R882wt/FLT3-ITD negatif hastalarda %62 oranında, p=0,025) ve nüksetme oranının arttığı görülmüştür.

**Sonuç:**

AML hastalarında allogeneik HKHN’den sonra bile, DNMT3A R882mut/FLT3-ITD pozitifliğinin kötü prognostik faktör olduğu sonucuna varılabilir.

## Introduction

Acute myeloid leukemia (AML) is considered a clonal disorder of the hematopoietic stem cells marked by proliferation of immature myeloid cells in the bone marrow (BM) or peripheral blood. Gene fusion, cell signaling abnormalities, and epigenetic modification affect the destination of hematopoietic stem cells and could lead to leukemogenesis [1,2].

Standard induction chemotherapy, which is a combination of cytarabine and anthracyclines, induces a high rate of complete remission (CR) in patients with AML; however, the rate of relapse is also high. This is more pronounced in elderly patients. Despite this fact, it is hoped that the outcome of patients is better when identifying and evaluating prognostic factors such as cytogenetics and molecular abnormalities [3]. A number of single-gene mutations have served for further risk stratification of AML patients. Risk stratification is one of the most important applications of molecular abnormalities, particularly in determining risk stratification after CR is achieved by induction therapy, and it is important because it prevents the referral of patients to hematopoietic stem cell transplantation (HSCT) centers [4]. As noted above, epigenetic modifications contribute to the formation of tumor cells. Epigenetic regulation refers to the modification of gene transcription and expression in such a way that the genetic code does not change [5].

DNA methylation is one of the most broadly studied mechanisms of epigenetic regulation. Methyltransferases are the key enzymes in the methylation process. DNMT3A belongs to the DNMTS family, which plays a significant role in adding methyl groups to cytosine residues in CpG islands. Actively transcribed genes exhibit a nonmethylated CpG profile. Cancer genomes are usually seen to have overall decrease in 5-methylcytosine, although DNA hypermethylation can be seen in some areas such as the promoter of tumor suppressor genes [5,6,7]. The exact mechanism of the function of DNMT3A mutations in the emergence of leukemia is unclear. Possible mechanisms include a change in enzyme catalytic properties and impaired binding to its ligand. DNMT3A has 23 exons and various mutations have been described to date. More than 60% of mutations are localized in the R882 hotspot region in the methyltransferase domain. DNMT3A mutations are predominantly heterozygous and strongly related to FLT3-ITD, IDH-1, and NPM-1 mutations. Differences in the incidence of DNMT3A mutations in AML patients were observed to range between 4.1% and 25% [8,9,10,11].

BM allogeneic HSCT is the only curative treatment for AML patients with intermediate or poor prognosis outcomes. DNMT3A and FLT3-ITD mutations have been found to be associated with adverse prognosis in patients with AML; however, few studies focused on the prognostic impact of these mutations in AML patients treated with allogeneic HSCT. The present study assessed the prognostic value of DNMT3A R882 and FLT3-ITD mutations in adult AML patients after allogeneic HSCT.

## Materials and Methods

### Patients

From August 2010 to September 2016, a total of 490 AML patients were referred to our center. DNA samples of 220 AML patients were available. Of those AML patients, 128 treated with allogeneic HSCT were enrolled in our study. Of these, 44 were female and 84 were male with a median age of 34 years (range: 15-67 years). Approval was obtained in writing from all patients in compliance with the Declaration of Helsinki and the ethical guidelines of Iran University of Medical Sciences.

The patients were diagnosed based on cytomorphology using the French-American-British (FAB) classification and immunophenotyping, and patients with AML-M3 with molecularly confirmed PML/RARA fusion gene were excluded from the study. All patients underwent allogeneic HSCT.

The source of hematopoietic stem cells for transplantation was peripheral blood, except for one patient who received BM, and donor types consisted of matched sibling donors (n=100), matched related donors (n=6), matched unrelated donors (n=7), and others (n=15).

### Treatment Regimens

The conditioning regimen was non-total body irradiation consisting of oral busulfan at 4 mg/kg (days -6 to day -3) and cyclophosphamide at 60 mg/kg (days -2 and day -1). Antithymocyte globulin (ATG) (r-ATG, 2.5 mg/kg, Thymoglobulin^®^, Genzyme) was used immediately before transplantation for 3 (in cases of matched related and haploidentical cases) or 2 (in other related cases) days. Graft-versus-host disease (GVHD) prophylaxis consisted of cyclosporine and methotrexate.

### Mutation Screenings

DNA was extracted from BM/peripheral blood mononuclear cells according to the standard salting-out extraction method. DNMT3A exon 23 was amplified by polymerase chain reaction (PCR) using forward (5¢-GTGTGGTTAGACGGCTTCC-3¢) and reverse (5¢-CTCTCCCACCTTTCCTCTG-3¢) primers. Polymerase chain reaction cycling conditions were the following: one cycle at 95 °C for 3 min, followed by 35 cycles of 94 °C for 30 s, 60 °C for 30 s, and 72 °C for 45 s, and then 72 °C for 7 min. Analysis of the PCR products was performed by electrophoresis on a 2% agarose gel using the BigDye Terminator v3.1 cycle sequencing kit (Applied Biosystems). The products of PCR were directly sequenced and an automated DNA sequencer (Applied Biosystems 3130 Genetic Analyzer) was employed. Comparing bidirectional sequence data to a normal reference sequence, positive mutations were recognized. Assessment of the NPM1 and FLT3-ITD mutations was performed by fragment analysis as previously described [12].

### Definition of Outcomes

The primary endpoints for survival analysis were overall survival (OS), calculated as the duration between the date of transplantation and death or last contact; relapse-free survival (RFS), calculated as time from the date of transplantation to first relapse; non-relapse mortality (NRM), calculated as time from transplantation to death from non-relapse causes; and cumulative incidence of relapse, defined as the time from allogeneic HSCT to the date of hematological relapse and considering death in remission as a competitive event. The grading of acute and chronic GVHD was based on criteria published previously [13].

### Statistical Analysis

Comparison of clinical characteristics of patients for continuous variables and categorical variables was done using the Mann-Whitney U test and Pearson’s chi-square test, respectively. The Kaplan-Meier method was applied to estimate OS and RFS and the log-rank test was used to compare groups. Multivariate Cox proportional hazards models were employed to assess OS and RFS. The cumulative incidence of NRM and relapse incidence (RI) were calculated considering competing risks. Death due to causes other than recurrence of disease and relapse were considered as competing events for relapse and NRM, respectively. A Fine-Gray proportional hazards regression model was used to evaluate the effects of covariates on RI and NRM. All variables with a p-value at or below 0.1 in the univariate analysis were entered in the multivariate analysis. Data analysis was performed using SPSS 19 and EZR software [14].

## Results

### Mutation Screening

In 20 patients (15.6%), DNMT3A exon 23 mutations were recognized. The R882 hotspot region harbors 19 mutations, including c.2645G>A, p.(R882H) (n=11); c.2644C>T, p.(R882C) (n=2); and c.2645G>C, p.( R882P) (n=6). One patient was found to be heterozygous for a Q905R missense mutation (Table 1). All mutations were heterozygous. In addition, the G884C synonymous variant was found in 3 patients.

Regarding presenting clinical features, no correlation was found between DNMT3A status and median age, sex, hemoglobin, or platelet and white blood cell (WBC) counts. Analysis of DNMT3A mutations was done with the FAB subtypes of AML. Nine of 20 DNMT3A exon 23 mutations belonged to M4/M5 of the FAB classification. The clinical characteristics and other molecular abnormalities of AML patients with mutated or unmutated DNMT3A are presented in Table 2. The prevalence of FLT3-ITD and NPM1 mutation was 28.1% and 18.8%, respectively. Among molecular aberrations, NPM1 mutations correlated with DNMT3A exon 23 mutations (p=0.014). A statistically significant correlation was observed between FLT3-ITD and NPM1 mutations (p=0.001).

### Clinical Outcomes Regarding Molecular Aberration Status after Allogeneic HSCT

**Figure 1:**
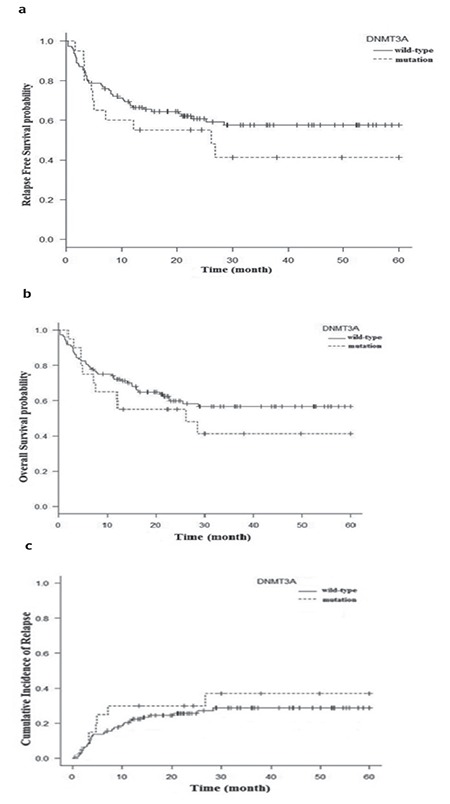
Survival curves of acute myeloid leukemia patients according to mutational status of DNMT3A: a) relapse-free survival, b) overall survival, c) cumulative incidence of relapse.

Analyses of OS and RFS were carried out with a 5-year follow-up period. Comparing AML patients with and without DNMT3A exon 23 mutations, no statistically significant difference was observed in OS (p=0.3) or RFS (p=0.29). The 5-year OS estimates were 41% in mutated DNMT3A patients versus 56% in AML patients without DNMT3A mutations and the 5-year RFS estimates were 41% versus 57%, respectively (Figure 1).

**Figure 2:**
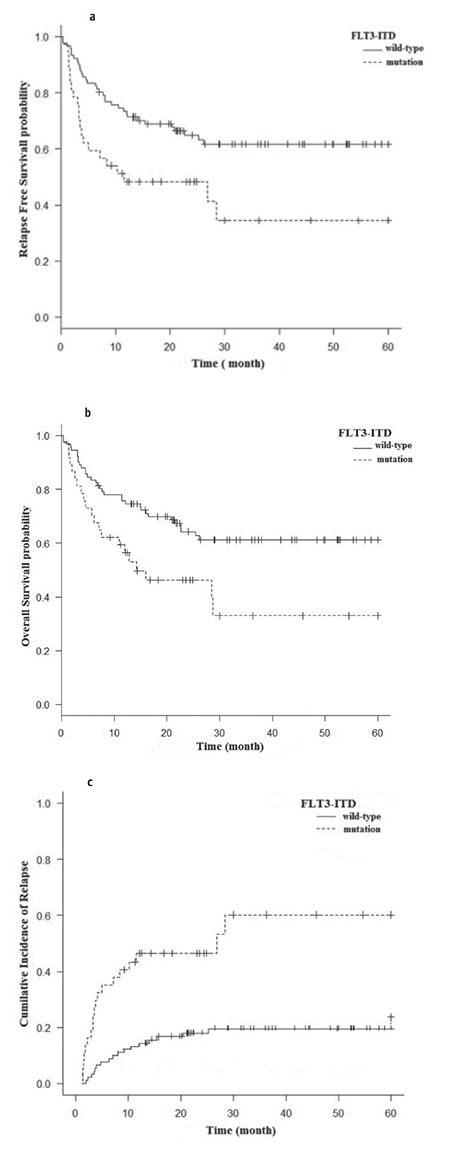
Survival curves of acute myeloid leukemia patients according to mutational status of FLT3-ITD: a) relapse-free survival, b) overall survival, c) cumulative incidence of relapse.

Analyses of OS and RFS were carried out regarding NPM1 and FLT3-ITD mutations. NPM1 had no impact on OS (p=0.83) or RFS (P=0.71). Regarding RI, a significant difference was observed between AML patients with and without NPM1 mutations (p=0.04). AML patients with FLT3-ITD mutations had lower OS (p=0.015) and RFS (p=0.012) compared to those without FLT3-ITD mutations (Figure 2).

The 5-year RI in the overall population was 30% with significant increase in RI regarding FLT3-ITD mutation (p=0.00003). Among other risk factors, the CR status at the time of hematopoietic cell transplantation was associated with higher RI (p=0.001). The FLT3-ITD mutation by 3.5-fold and the CR status at allogeneic HSCT by 2.5-fold triggered an increase in the risk of relapse.

The cumulative incidence of NRM within 5 years was 19.3%. Neither DNMT3A exon 23 mutations nor the majority of risk factors were found to be in association with NRM. Only in multivariate analysis did chronic GVHD increase the risk of NRM by 5.2-fold (p=0.007, HR=5.23; 95% CI: 1.66-16.5).

**Figure 3:**
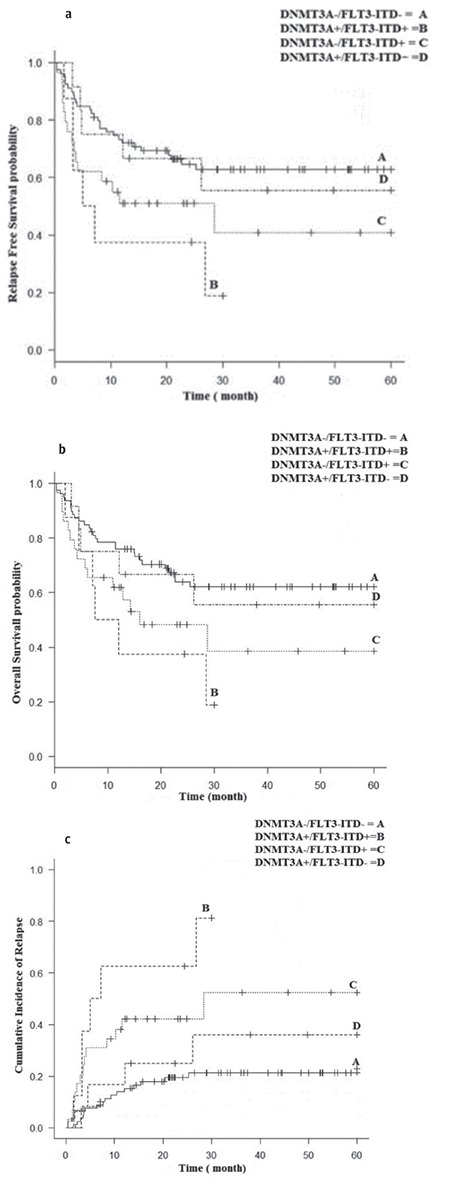
Survival curves of acute myeloid leukemia patients according to mutational status of DNMT3A/FLT3-ITD: a) relapsefree survival, b) overall survival, c) cumulative incidence of relapse.

In the next step, AML patients based on DNMT3A and FLT3-ITD mutations were divided into four groups: group A (DNMT3A R882wt /FLT3-ITDneg), group B (DNMT3A R882mut /FLT3-ITDpos), group C (DNMT3A R882wt/FLT3-ITDpos), and group D (DNMT3A R882mut/FLT3-ITDneg). Detailed information about these groups is summarized in Table 3. WBC counts (p=0.003) and CR status at allogeneic HSCT (p=0.022) were statistically significant among groups. Patients with DNMT3Awt/FLT3-ITDpos (group C) had the highest WBC counts compared with other groups. Considering the CR status (p=0.01) and WBC count (p=0.004), the differences were statistically significant in the DNMT3Awt/FLT3-ITDpos group (group C) compared with DNMT3Awt/FLT3-ITDneg (group A). The cumulative incidence of relapse, OS, and RFS rates were compared according to these groups. In univariate analysis, the differences in OS and RFS between AML patients with coexistence of DNMT3Amut/FLT3-ITDpos (group B) and those with DNMT3Awt/FLT3-ITDneg (group A) were statistically significant. The DNMT3Amut/FLT3-ITDpos patients (group B) had the worst OS (p=0.025) and RFS (p=0.011) compared with other groups, revealing a higher RI rate (p=0.0002) (Table 4, Figure 3).

### Multivariate Analyses for OS, RFS, and RI

Multivariate analyses for RFS, OS, and RI were carried out regarding the CR condition (CR1 or CR≥2), the interval from CR1 to transplantation, FLT3-ITD mutation, DNMT3Amut/FLT3-ITDpos, and DNMT3Awt/FLT3-ITDpos. The FLT3-ITD mutation (p=0.03, HR=1.84; 95% CI: 1.05-3.24) and CR status (p=0.04, HR=1.78; 95% CI: 1.02-3.13) were independent factors of inferior survival after allogeneic HSCT. Regarding RI, CR status to transplantation (p=0.0049, HR= 2.52; 95% CI: 1.32-4.8) and FLT3-ITD mutation (p=0.00015, HR=3.49; 95% CI: 1.83-6.68) were significant independent prognostic factors for relapse (Table 5).

## Discussion

Although DNMT3A mutations were recognized as driver gene mutations in adults with AML, their roles in leukemogenesis remain poorly understood. In the past decade, however, DNMT3A mutations have been attracting much attention as markers for risk stratification in AML patients [15].

The present study showed that DNMT3A mutations occur in 15.6% (20/128) of AML patients, predominantly in patients with NPM1 aberrations. The dominant mutation in the study population being located at hotspot region R882 is in agreement with previous studies [16,17].

The present study finds that DNMT3A R882 mutations are not related to inferior survival in AML patients after allogeneic HSCT. It could be argued that allogeneic HSCT ameliorates the clinical consequences of AML cases with DNMT3A R882 mutations. In the present study, no significant difference was found in OS, RFS, or RI between cases with DNMT3A mutations and cases with wild-type DNMT3A.

Several studies with controversial results have been conducted on the prognostic impact of DNMT3A mutations in AML patients. Some studies revealed a statistically significant difference in OS between mutated and unmutated DNMT3A patients, with worse OS in the mutated cases [16,18,19,20].

Metzeler et al. [21] showed that DNMT3A mutations are on one hand related to inferior survival in AML patients and on the other hand modify the prognostic effect of mutated NPM1. They also found that different types of DNMT3A mutations had no effect on patient outcomes. Yuan et al. [22], in a meta-analysis of DNMT3A R882 mutations in AML patients consisting of eight studies with 4474 AML cases with 694 AML patients with DNMT3A R882 mutations, verified significantly reduced RFS and OS in AML patients with DNMT3A R882 mutations.

Focusing on the characteristics and effects of DNMT3A R882 mutation in AML patients with or without NPM1 and FLT3 mutations, Dushyant et al. analyzed 174 AML patients with normal cytogenetics. They noticed that DNMT3A mutations in the cytogenetically normal AML patients compared to NPM1- and FLT3-mutated patients (p=0.067 and p=0.065, respectively) were related to remarkably shorter OS and progression-free survival [23].

However, few studies, as mentioned in the following text, have been designed to determine the prognostic effect of DNMT3A mutations in AML patients treated with allogeneic HSCT.

Consistent with our results, Xu et al. [24] compared the outcomes of 55 DNMT3A (mut) patients who underwent allogeneic HSCT (23 cases) or received chemotherapy (32 cases) for consolidation. They observed a significant difference in 3-year OS and DFS between the chemotherapy group and the allogeneic HSCT group. The authors concluded that DNMT3A mutations act as an unfavorable prognostic factor in AML patients with normal cytogenetics and allogeneic HSCT improves survival in DNMT3A mutation-positive AML patients. The median OS in wild-type DNMT3A patients was greater compared to mutated patients but the difference was not statistically significant (p=0.151). No significant difference was seen in DFS between these groups of patients (p=0.304) [24].

Ahn et al. [25] described DNMT3A R882mut and FLT3-ITDpos as both unfavorable prognostic markers for OS and significant risk factors for relapse and event-free survival. They declared that patients with coexistence of DNMT3A R882 and FLT3-ITD mutations had worse OS, worse event-free survival, and higher relapse rates compared with other mutations. Indeed, DNMT3A R882mut/FLT3-ITDpos status was a significant prognostic marker for poor clinical outcome with increasing RI rates even after HSCT.

Tang et al. [26] verified that DNMT3A R882 mutations confer inferior survival in AML patients. Their results also indicated that coexistence of FLT3-ITD and DNMT3A R882 mutations was an independent factor for adverse prognosis after allogeneic HSCT.

Contrary to our results, the findings of Ahn et al. [25] and Tang et al. [26] considered DNMT3A R882 as an unfavorable prognostic indicator in AML patients treated with allogeneic HSCT. The reason for this discrepancy between the results is not clear, but unknown cooperating genetic aberrations may be involved. Several studies have shown that allogeneic HSCT cannot abrogate the unfavorable effect of FLT3-ITD in AML patients [27,28]. Therefore, in the next step, we analyzed the surveillance factors of OS, RFS, and RI in FLT3-ITDpos/DNMT3A R882mut AML patients and encountered the worst condition compared with other groups. These results are consistent with the findings of the above-mentioned studies [25,26].

Our data also highlighted that CR status prior to transplantation was more obvious than other risk factors in delineating risk of relapse. In more advanced disease stages (CR2, CR3), more RI occurs. From this point of view, our data are in accordance with previous studies [29,30].

### Study Limitations

The limitations of this research include the following: first, a relatively small sample size was used; second, the analysis was limited to exon 23 of the DNMT3A gene; and third, there is an absence of cytogenetic findings. Hence, caution should be taken in the interpretation of the results of the present study.

The initial goal of allogeneic HSCT is to improve hematological disorders while minimizing residual disease as much as possible. To achieve this goal, the patient should be supported through a conditioning regimen and its associated complications such as GVHD while avoiding relapse. ATG can reduce the risk of GVHD, although ATG formulation (dose and type), donor type, and other medications used for GVHD prophylaxis affect the outcome of allogeneic HSCT [31]. In the present study, in order to avoid reducing the number of patients, the role of ATG and donor source were ignored.

Allogeneic HSCT is a pragmatic treatment option for relapsed and/or refractory AML patients; however, it seems that allogeneic HSCT cannot override the inferior outcomes conferred by the coexistence of DNMT3Amut/FLT3-ITDpos. Our increased knowledge of genetic and epigenetic alterations in AML has triggered the emergence of new medicines such as CPX-351, FLT3 inhibitors, and epigenetic modifiers. Indeed, it is necessary to accompany molecularly targeted therapy with allogeneic HSCT for poor prognosis in AML patients.

## Conclusion

Based on the findings of the present study, DNMT3A R882 mutations seem not to affect the clinical outcomes of AML patients undergoing allogeneic HSCT. In contrast, allogeneic HSCT probably improves the clinical outcomes of AML patients with DNMT3A R882 mutations. When DNMT3A R882 mutations were accompanied by FLT3-ITD mutations (DNMT3A R882mut/FLT3-ITDpos), OS was significantly reduced, even after allogeneic HSCT. Indeed, FLT3-ITD is a significant negative prognostic indicator compared with the DNMT3A R882 mutation. Further studies are required to better explain a rationale for the integration of DNMT3A R882mut/FLT3-ITDpos status in the treatment decisions of AML patients.

## Figures and Tables

**Table 1:**
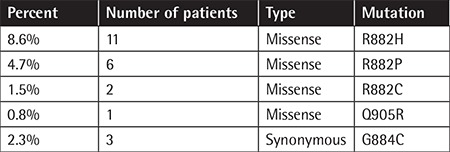
Frequency of DNMT3A exon 23 mutations in acute myeloid leukemia patients.

**Table 2:**
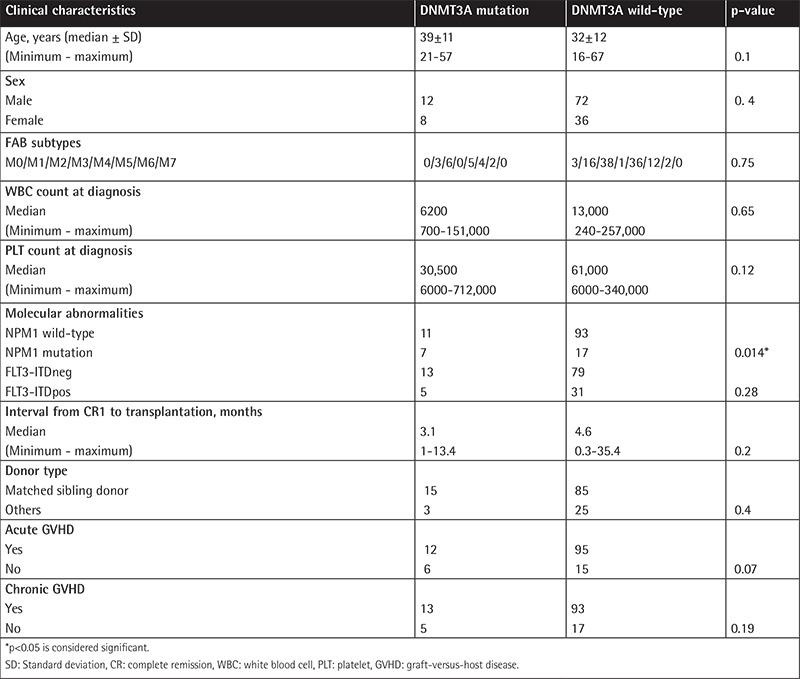
Clinical characteristics of acute myeloid leukemia patients according to DNMT3A status.

**Table 3:**
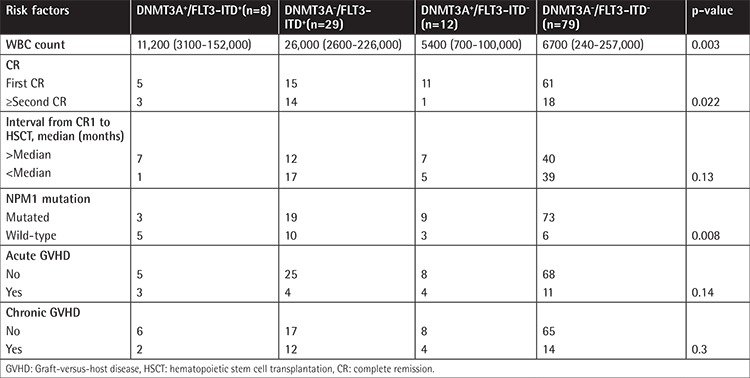
Clinical characteristics of acute myeloid leukemia patients according to DNMT3A R882/FLT3-ITD groups.

**Table 4:**
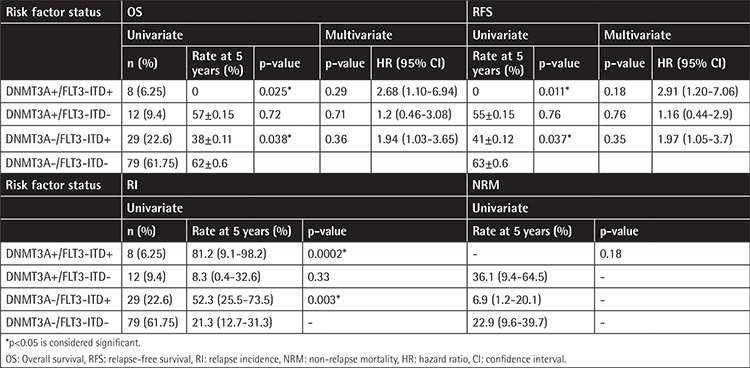
Univariate and multivariate analysis of surveillance factors according to DNMT3A R882/FLT3-ITD groups.

**Table 5:**
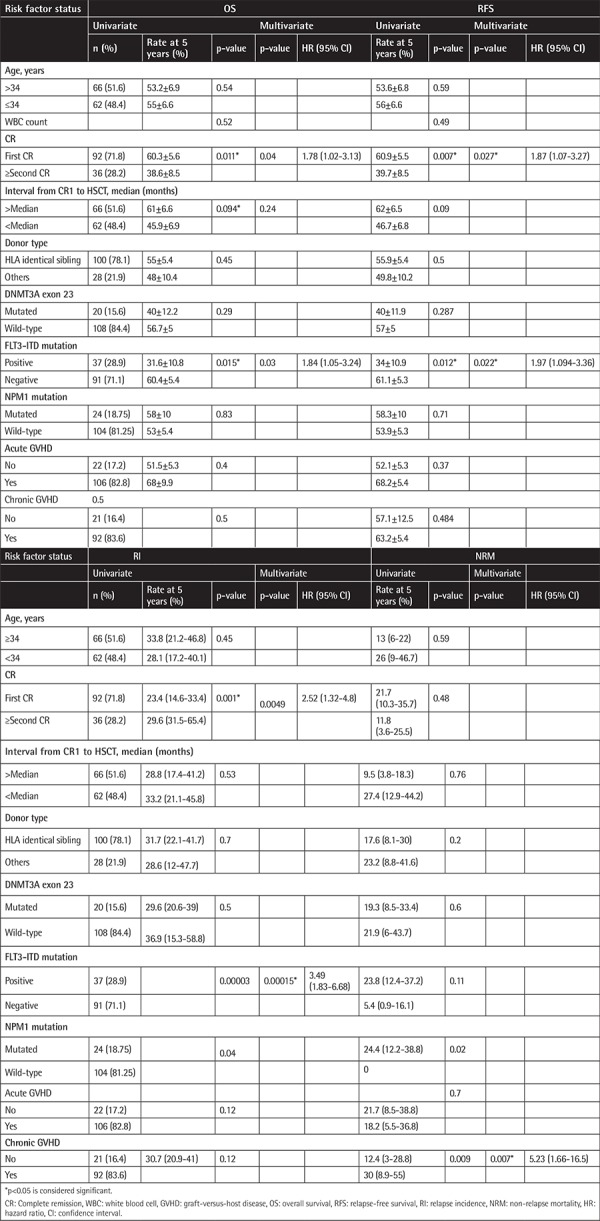
Univariate and multivariate analysis of surveillance factors according to risk factor status.

## References

[1] Loghavi S, Zuo Z, Ravandi F, Kantarjian HM, Bueso-Ramos C, Zhang L, Singh RR, Patel KP, Medeiros LJ, Stingo F, Routbort M, Cortes J, Luthra R, Khoury JD (2014). Clinical features of de novo acute myeloid leukemia with concurrent *DNMT3A*, *FLT3* and *NPM1* mutations. J Hematol Oncol.

[2] Prada-Arismendy J, Arroyave JC, Röthlisberger S (2017). Molecular biomarkers in acute myeloid leukemia. Blood Rev.

[3] Döhner H, Estey E, Grimwade D, Amadori S, Appelbaum FR, Büchner T, Dombret H, Ebert BL, Fenaux P, Larson RA, Levine RL, Lo-Coco F, Naoe T, Niederwieser D, Ossenkoppele GJ, Sanz M, Sierra J, Tallman MS, Tien HF, Wei AH, Löwenberg B, Bloomfield CD (2017). Diagnosis and management of AML in adults: 2017 ELN recommendations from an international expert panel. Blood.

[4] Wang ML, Bailey NG (2015). Acute myeloid leukemia genetics: risk stratification and implications for therapy. Arch Pathol Lab Med.

[5] Oki Y, Issa JP (2010). Epigenetic mechanisms in AML–a target for therapy. Cancer Treat Res.

[6] Shih AH, Abdel-Wahab O, Patel JP, Levine RL (2012). The role of mutations in epigenetic regulators in myeloid malignancies. Nat Rev Cancer.

[7] Suzuki MM, Bird A (2008). DNA methylation landscapes: provocative insights from epigenomics. Nat Rev Genet.

[8] Challen GA, Sun D, Jeong M, Luo M, Jelinek J, Berg JS, Bock C, Vasanthakumar A, Gu H, Xi Y, Liang S, Lu Y, Darlington GJ, Meissner A, Issa JP, Godley LA, Li W, Goodell MA (2012). Dnmt3a is essential for hematopoietic stem cell differentiation. Nat Genet.

[9] Jia D, Jurkowska RZ, Zhang X, Jeltsch A, Cheng X (2007). Structure of Dnmt3a bound to Dnmt3L suggests a model for de novo DNA methylation. Nature.

[10] Pezzi A, Moraes L, Valim V, Amorin B, Melchiades G, Oliveira F, da Silva MA, Matte U, Pombo-de-Oliveira MS, Bittencourt R, Daudt L, Silla L (2012). DNMT3A mutations in patients with acute myeloid leukemia in South Brazil. Adv Hematol.

[11] Russler-Germain DA, Spencer DH, Young MA, Lamprecht TL, Miller CA, Fulton R, Meyer MR, Erdmann-Gilmore P, Townsend RR, Wilson RK, Ley TJ (2014). The R882H DNMT3A mutation associated with AML dominantly inhibits wild-type DNMT3A by blocking its ability to form active tetramers. Cancer Cell.

[12] Ghasemi A, Nadali F, Chahardouli B, Ghandforosh NA, Zadeh AG, Rostami S (2014). Study of correlation between SFRP-1 and SFRP-2 hypermethylation with relapse, complete remission, genetic mutations of FLT3-ITD and NPM1 and immunophenotypes of leukemic cells in patients with de novo acute myeloblastic leukemia. Journal of Hematology.

[13] Glucksberg H, Storb R, Fefer A, Buckner CD, Neiman PE, Clift RA, Lerner KG, Thomas ED (1974). Clinical manifestations of graft-versus-host disease in human recipients of marrow from HL-A-matched sibling donor. Transplantation.

[14] Kanda Y (2013). Investigation of the freely available easy-to-use software ‘EZR’ for medical statistics. Bone Marrow Transplant.

[15] Metzeler KH, Herold T, Rothenberg-Thurley M, Amler S, Sauerland MC, Görlich D, Schneider S, Konstandin NP, Dufour A, Bräundl K, Ksienzyk B, Zellmeier E, Hartmann L, Greif PA, Fiegl M, Subklewe M, Bohlander SK, Krug U, Faldum A, Berdel WE, Wörmann B, Büchner T, Hiddemann W, Braess J, Spiekermann K;, AMLCG Study Group (2016). Spectrum and prognostic relevance of driver gene mutations in acute myeloid leukemia. Blood.

[16] Renneville A, Boissel N, Nibourel O, Berthon C, Helevaut N, Gardin C, Cayuela JM, Hayette S, Reman O, Contentin N, Bordessoule D, Pautas C, Botton Sd, Revel Td, Terre C, Fenaux P, Thomas X, Castaigne S, Dombret H, Preudhomme C (2012). Prognostic significance of DNA methyltransferase 3A mutations in cytogenetically normal acute myeloid leukemia: a study by the Acute Leukemia French Association. Leukemia.

[17] Ostronoff F, Othus M, Ho PA, Kutny M, Geraghty DE, Petersdorf SH, Godwin JE, Willman CL, Radich JP, Appelbaum FR, Stirewalt DL, Meshinchi S (2013). Mutations in the *DNMT3A* exon 23 independently predict poor outcome in older patients with acute myeloid leukemia: a SWOG report. Leukemia.

[18] Hou HA, Kuo YY, Liu CY, Chou WC, Lee MC, Chen CY, Lin LI, Tseng MH, Huang CF, Chiang YC, Lee FY, Liu MC, Liu CW, Tang JL, Yao M, Huang SY, Ko BS, Hsu SC, Wu SJ, Tsay W, Chen YC, Tien HF (2012). DNMT3A mutations in acute myeloid leukemia-stability during disease evolution and the clinical implication. Blood.

[19] Ley TJ, Ding L, Walter MJ, McLellan MD, Lamprecht T, Larson DE, Kandoth C, Payton JE, Baty J, Welch J, Harris CC, Lichti CF, Townsend RR, Fulton RS, Dooling DJ, Koboldt DC, Schmidt H, Zhang Q, Osborne JR, Lin L, O’Laughlin M, McMichael JF, Delehaunty KD, McGrath SD, Fulton LA, Magrini VJ, Vickery TL, Hundal J, Cook LL, Conyers JJ, Swift GW, Reed JP, Alldredge PA, Wylie T, Walker J, Kalicki J, Watson MA, Heath S, Shannon WD, Varghese N, Nagarajan R, Westervelt P, Tomasson MH, Link DC, Graubert TA, DiPersio JF, Mardis ER, Wilson RK (2010). DNMT3A mutations in acute myeloid leukemia. N Engl J Med.

[20] Thol F, Damm F, Lüdeking A, Winschel C, Wagner K, Morgan M, Yun H, Göhring G, Schlegelberger B, Hoelzer D, Lübbert M, Kanz L, Fiedler W, Kirchner H, Heil G, Krauter J, Ganser A, Heuser M (2011). Incidence and prognostic influence of DNMT3A mutations in acute myeloid leukemia. J Clin Oncol.

[21] Metzeler KH, Herold T, Rothenberg-Thurley M, Amler S, Sauerland C, Schneider S, Konstandin NP, Dufour AM, Bräundl K, Ksienzyk B, Zellmeier E, Hartmann L, Greif PA, Fiegl M, Subklewe MS, Bohlander SK, Krug U, Berdel WE, Wörmann B, Büchner T, Faldum A, Hiddemann W, Braess J, Spiekermann K (2015). DNMT3A mutations associate with shorter survival and modulate the prognostic impact of mutated NPM1: an analysis based on comprehensive mutational screening of 660 AML patients treated on German AML Cooperative Group (AMLCG) trials. Blood.

[22] Yuan XQ, Peng L, Zeng WJ, Jiang BY, Li GC, Chen XP (2016). DNMT3A R882 mutations predict a poor prognosis in AML: a meta-analysis from 4474 patients. Medicine (Baltimore).

[23] Kumar D, Mehta A, Panigrahi MK, Nath S, Saikia KK (2018). DNMT3A (R882) mutation features and prognostic effect in acute myeloid leukemia in coexistent with NPM1 and FLT3 mutations. Hematol Oncol Stem Cell Ther.

[24] Xu Y, Sun Y, Shen H, Ding L, Yang Z, Qiu H, Sun A, Chen S, Wu D (2015). Allogeneic hematopoietic stem cell transplantation could improve survival of cytogenetically normal adult acute myeloid leukemia patients with DNMT3A mutations. Am J Hematol.

[25] Ahn JS, Kim HJ, Kim YK, Lee SS, Jung SH, Yang DH, Lee JJ, Kim NY, Choi SH, Jung CW, Jang JH, Kim HJ, Moon JH, Sohn SK, Won JH, Kim SH, Kim DD (2016). DNMT3A R882 mutation with FLT3-ITD positivity is an extremely poor prognostic factor in patients with Normal-Karyotype acute myeloid leukemia after allogeneic hematopoietic cell transplantation. Biol Blood Marrow Transplant.

[26] Tang S, Shen H, Mao X, Dai H, Zhu X, Xue S, Ding Z, Lu J, Wu D, Tang X (2017). FLT3-ITD with DNMT3A R882 double mutation is a poor prognostic factor in Chinese patients with acute myeloid leukemia after chemotherapy or allogeneic hematopoietic stem cell transplantation. Int J Hematol.

[27] Brunet S, Martino R, Sierra J (2013). Hematopoietic transplantation for acute myeloid leukemia with internal tandem duplication of FLT3 gene (FLT3/ITD). Curr Opin Oncol.

[28] Koreth J, Schlenk R, Kopecky KJ, Honda S, Sierra J, Djulbegovic BJ, Wadleigh M, DeAngelo DJ, Stone RM, Sakamaki H, Appelbaum FR, Döhner H, Antin JH, Soiffer RJ, Cutler C (2009). Allogeneic stem cell transplantation for acute myeloid leukemia in first complete remission: systematic review and meta-analysis of prospective clinical trials. JAMA.

[29] Warlick ED, Peffault de Latour R, Shanley R, Robin M, Bejanyan N, Xhaard A, Brunstein C, Sicre de Fontbrune F, Ustun C, Weisdorf DJ, Socie G (2015). Allogeneic hematopoietic cell transplantation outcomes in acute myeloid leukemia: similar outcomes regardless of donor type. Biol Blood Marrow Transplant.

[30] Chevallier P, Labopin M, Cornelissen J, Socie G, Rocha V, Mohty M;, ALWP of EBMT (2011). Allogeneic hematopoietic stem cell transplantation for isolated and leukemic myeloid sarcoma in adults: a report from the Acute Leukemia Working Party of the European group for Blood and Marrow Transplantation. Haematologica.

[31] Kekre N, Antin JH (2017). ATG in allogeneic stem cell transplantation: standard of care in 2017? Counterpoint. Blood Adv.

